# Development of Encapsulation Strategies and Composite Edible Films to Maintain Lactoferrin Bioactivity: A Review

**DOI:** 10.3390/ma14237358

**Published:** 2021-11-30

**Authors:** Inés Abad, Celia Conesa, Lourdes Sánchez

**Affiliations:** 1 Departamento de Producción Animal y Ciencia de los Alimentos, Facultad de Veterinaria, Universidad de Zaragoza, 50013 Zaragoza, Spain; inesabadc@unizar.es (I.A.); cconesa@unizar.es (C.C.); 2 Instituto Agroalimentario de Aragón (IA2), Universidad de Zaragoza-CITA, 50013 Zaragoza, Spain

**Keywords:** lactoferrin, nanocarriers, nanoparticles, microparticles, encapsulation, edible films, active films, active packaging, milk proteins, polysaccharides, technological treatments, gastrointestinal tract, targeted delivery, food technology

## Abstract

Lactoferrin (LF) is a whey protein with various and valuable biological activities. For this reason, LF has been used as a supplement in formula milk and functional products. However, it must be considered that the properties of LF can be affected by technological treatments and gastrointestinal conditions. In this article, we have revised the literature published on the research done during the last decades on the development of various technologies, such as encapsulation or composite materials, to protect LF and avoid its degradation. Multiple compounds can be used to conduct this protective function, such as proteins, including those from milk, or polysaccharides, like alginate or chitosan. Furthermore, LF can be used as a component in complexes, nanoparticles, hydrogels and emulsions, to encapsulate, protect and deliver other bioactive compounds, such as essential oils or probiotics. Additionally, LF can be part of systems to deliver drugs or to apply certain therapies to target cells expressing LF receptors. These systems also allow improving the detection of gliomas and have also been used for treating some pathologies, such as different types of tumours. Finally, the application of LF in edible and active films can be effective against some contaminants and limit the increase of the natural microbiota present in meat, for example, becoming one of the most interesting research topics in food technology.

## 1. Introduction

Lactoferrin (LF) is an iron-binding glycoprotein present in the secretions of mammalian species. It was first isolated from milk, and afterwards, it was found in mucosal surfaces, specific granules of leukocytes and several secretory fluids including tears, saliva, seminal and nasal secretions [1]. LF plays important roles, such as modulation of iron uptake and release by the intestinal mucosal cells in the suckling newborn; antimicrobial activity against bacteria, virus, fungus and parasites; antioxidant activity in several systems and regulation of immunity and cellular growth [2]. When LF is intended to be used as an ingredient in some functional products, it is essential to know the effect that technological treatments exert on its activity [3].

LF is highly unstable under the conditions of the stomach and intestines due to the gastric low pH and the high protease activity present in both compartments that cause conformational changes and loss of activity to this protein [4,5,6]. Taking this susceptibility into account, it is important to achieve LF stabilization by using some technologies, such as encapsulation or composite materials, to avoid its degradation along the gastrointestinal tract (GIT) [7]. Apart from the interest in encapsulating LF to preserve its beneficial biological activities, this protein is used as a component in complexes, nanoparticles, hydrogels and emulsions to encapsulate, protect and deliver other bioactive compounds [8]. In addition, LF is also being investigated to be part of systems to deliver drugs or to apply certain therapies to target cells expressing LF receptors on their surface [9].

Encapsulation techniques have been developed in the last decades to preserve bioactive compounds incorporated into pharmaceutics, food and cosmetic products [10]. Microencapsulation is defined as a process in which very small particles or droplets are surrounded by a coating or are embedded in homogeneous or heterogeneous matrices. This process is intended to preserve bioactive compounds from inactivation, which can be caused by certain conditions and interactions with the environment where those compounds are intended to exert their activity [11]. Nanocarrier systems have many advantages, such as improving the bioavailability and solubility of bioactive compounds, enhancing their residence time and stability in the GIT and giving them a better ability to enter and permeate tissues and cells [12].

Nowadays, research in edible films is a relevant emerging area of study because the reduction of plastic waste is of high priority at the global level. Edible films and coatings avoid the desiccation of the food product, increasing its structure resistance and controlling gas exchange [13,14]. Furthermore, edible films and coatings can contain and deliver active ingredients that add extra properties to them, such as antioxidant and antimicrobial properties, by incorporating bioactive compounds like LF [14].

The development of nanotechnology has produced great changes in many areas, such as the way to administer some therapies or the design of particles that can be directed to specific targets. The present review covers the current emerging technologies that can be used to preserve the biological activity of LF in different systems. This report is not comprehensive, as the studies on LF are numerous and it is impossible to cover all of them; however, the articles revised here are representative of the research performed on this field in recent years. We have focused on the mechanisms to encapsulate LF to preserve its integrity, the use of LF to decorate particles with the aim to improve the delivery of compounds to specific cells and finally, on the use of LF in active films for food preservation.

## 2. Materials and Methods

### 2.1. Search Strategy

The three authors performed the electronic search from 1995 to September 2021, using Medline (PubMed) and ScienceDirect. The search strategy applied was a combination of medical subject headings (MeSH), terms and free text words, including the following keywords individually or combined: lactoferrin, nanocarriers, nanoparticles, microparticles, encapsulation, edible films, active films, active packaging, milk proteins, whey proteins, caseins, polysaccharides, alginate, chitosan, pectin, gums, chondroitin sulphate and galactomannans.

**Figure 1:**
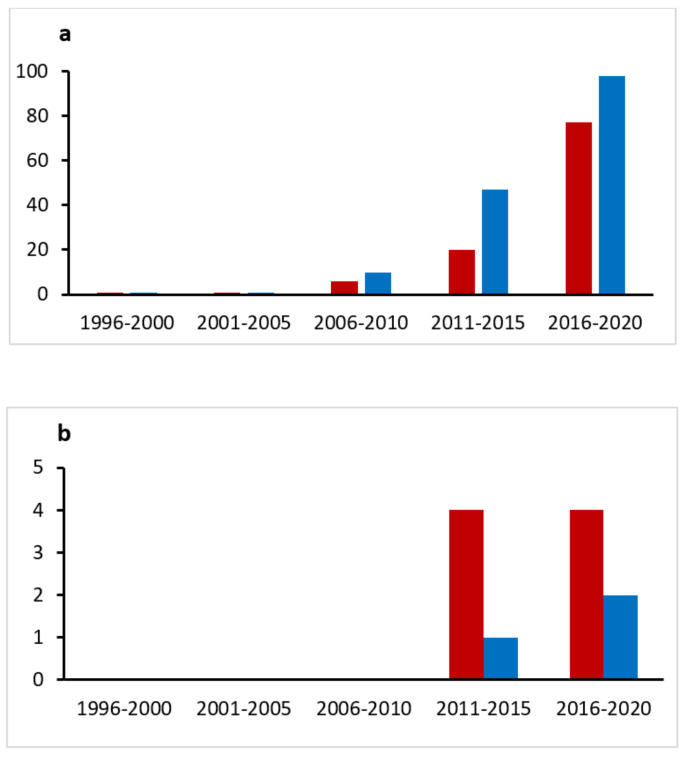
Graphs representing the publications found by combining the term lactoferrin with (**a**): nanoparticles, microparticles or encapsulation; (**b**): edible films, active films or active packaging, in PubMed (blue bars) and ScienceDirect (red bars) shown with years.

Review articles and the different research articles found were also sources of references to locate other articles. The procedure used for selecting the articles and type of articles finally included in the review was discussed by all the authors and finally, 153 articles were revised and included in the review. Furthermore, the evolution in the number of publications related to some terms used in the search was analysed over the course of twenty-five years, as is represented in Figure 1a,b.

### 2.2. Selection of Studies

The review process was divided into three major sections to be revised initially by each of the three authors (I.A., C.C. and L.S.), and afterwards, all authors revised the three sections. The selection of the articles was first screened for relevance by reading the abstracts. Additionally, the relevant references of the selected articles were searched manually. The following exclusion and inclusion criteria were used to select the obtained articles:

#### 2.2.1. Inclusion Criteria

1. The article includes a description of the procedure to make LF composites;
2. The article includes applications of LF composites related to medicine;
3. The article includes applications of LF composites related to foods;
4. Review articles that help to identify articles related to the topics for review.

#### 2.2.2. Exclusion Criteria

1. Articles not providing detailed information on the preparation of LF composites;
2. Articles not providing detailed information on the applications of LF composites;
3. Articles published before the year 1999.

## 3. Results

### 3.1. Encapsulation of Lactoferrin

The selection of appropriate materials for encapsulating bioactive compounds is essential. These materials should be chemically compatible, non-reactive with the encapsulated substance and are expected to show certain properties, such as strength, flexibility, impermeability and stability, which are required for their application [15,16].

There are various food biopolymers, including polysaccharides, lipids, proteins, and their conjugates used to develop a wide range of nanocarriers. These are designed to protect, entrap, encapsulate and control the delivery of bioactive compounds and nutraceuticals [12]. The systems based on lipid carriers can be classified as nanoemulsions, solid lipid nanoparticles, nanostructured lipid carriers, nanoliposomes, micelles and nanosuspensions [17]. Polysaccharide-based nanocarriers also include polymer nanoparticles, polymeric micelles and inclusion complexes [12]. However, nanocarriers based on food proteins can be prepared with different structures, including nanoparticles, hollow nanoparticles, nanohydrogels, heat-induced nanofibrillar aggregates, electrospun nanofibers and tubular nanostructures [18].

Food proteins normally used to construct nanocarriers can derive from animals and plants, such as whey, soy, egg, corn proteins, gelatin and bovine serum albumin (BSA), among others. The nanocarriers produced with those proteins are adequate for the delivery of both hydrophobic and hydrophilic bioactive ingredients and can be included in products for nutrition, drugs or can be directed to apply several therapies [12].

**Figure 2:**
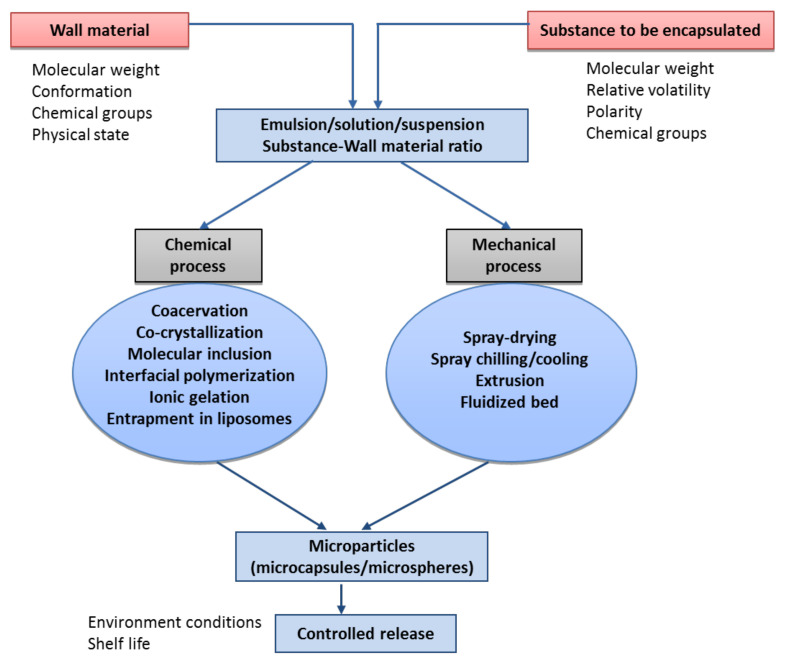
A schematic illustration of different processes of encapsulation used in food and flavour industries (adapted from Madene et al. [19]).

Encapsulation can be conducted by chemical or mechanical procedures, as shown in Figure 2. Chemical encapsulation can be done by simple and complex coacervation, co-crystallization, interfacial polymerization, ionic gelation, entrapment in liposomes, molecular inclusion and ionic gelation plus electrostatic interactions. The mechanical procedures are spray-drying, freeze/cold-drying, extrusion and fluidized bed [19].

The complex coacervates are generated by an encapsulation method that usually consists of three stages: emulsification, coacervation and cross-linking. The complex coacervation is a liquid–liquid phase separation that occurs when there is an interaction, mainly electrostatic, between biopolymers of opposite charges, the interaction being influenced by pH. The polymer matrix, considered as the wall, mainly made up of positively charged proteins and negatively charged polymers surrounds the bioactive compound, considered as the core [20]. The polymer-based coacervates have many applications as food and biomaterial ingredients or encapsulants in personal care and functional foods. Recently, there has been an increase in the interest to study heteroprotein complex coacervates, which are a special case of coacervates in which the dense phase is formed by at least two different proteins with opposite charges [21].

The potential benefits of bioactive compounds, also known as nutraceuticals, are not optimally achieved because of their low and/or variable bioavailability [22]. This availability is a measure of the bioefficiency of bioactive compounds and is the result of processes that those components undergo within four gastrointestinal segments: the mouth, stomach, small intestine and large intestine [23].

The factors that mainly affect the bioavailability of encapsulated bioactive compounds are the composition of nanocarriers and loading capacity, size and surface. For example, nanocarriers based on starch and proteins can be hydrolysed by amylases and proteases, while other materials, such as resistant starch, pectin and alginate are resistant to enzymes and pH, and are attacked only by the colonic microbiota [23]. However, the release of bioactive compounds from lipid-based nanocarriers is favoured by the action of lipase in the mouth, stomach and intestines, which breaks down triacylglycerides into free fatty acids and monoacylglycerides, contributing, with the bile salts, to the solubilisation of bioactives into mixed micelles [24]. Furthermore, the bioavailability of many bioactive components depends on the food matrix that is coingested with them, in the case that those are contained in a food product or in some nutraceuticals.

#### 3.1.1. Encapsulation of Lactoferrin with Milk Proteins

Milk proteins have a rich diversity of physicochemical characteristics and biodegradable properties, which make them appealing for different food and pharmaceutical applications [25]. Proteins are natural vehicles for bioactive molecules, thanks to their structural and physicochemical properties that facilitate their functionality in delivery systems. For oral delivery applications, the biocompatibility of milk proteins is usually positive [26], although it is necessary to consider that a small portion of the population is allergic to some milk proteins.

- Whey proteins

Generally, whey proteins and LF are electrostatically attracted to each other over a range of pH values due to their different isoelectric points (pI), and so they can be assembled in various interfacial structures, such as single, double and mixed layers in emulsions [27]. In that sense, Anema and de Kruif [28] assayed the mixture of LF with the anionic protein β-lactoglobulin (β-Lg) at a range of pH and mixing ratios. Complexation was monitored through turbidity and zeta potential measurements, and the formation of complex coacervates was observed. Complexes were formed in the pH region of 5–7.3, and at the maximum turbidity, the complexes were neutrally charged. Moreover, although the charge ratios of LF/β-Lg varied with pH, LF/β-Lg complexes were found to have a constant stoichiometry of 1:3 at all pHs due to charge neutralization. With the addition of NaCl, the complexation diminished and disappeared at a salt concentration of about 100 mM.

Chapeau et al. [29] showed that electrostatic complexes formed between LF and β-Lg can be used to successfully encapsulate vitamin B9 at its recommended daily intake levels. They identified two types of B9/LF/β-Lg co-assembly: aggregates (at low and high protein concentrations) and heteroprotein coacervates (at intermediate protein concentrations), both exhibiting different kinetics and stability over time. The B9/LF/β-Lg coacervates showed high entrapment of B9 (from 6 to 16 mg B9/g protein), which means that a few milligrams of these coacervates would be enough to cover the recommended daily intake of this vitamin. The same research group also demonstrated the scale-up of this system, confirming the efficiency of this type of biocarrier for developing natural functional foods [30].

Whey protein isolate (WPI) is obtained from the main by-product of cheese production and mainly consists of β-Lg and α-lactalbumin (α-La). Irreversible denaturation and aggregation of whey proteins occur at heating temperatures higher than 70 °C, and the denatured proteins interact with each other, forming a gel network. WPI has a strong binding affinity for LF as it can cross-link to form a stable protein complex with enhanced elasticity and gel strength under various heating conditions, as reported by Li and Zhao [31]. These researchers showed that the addition of LF into WPI gels increased the gel strength at the two pHs tested of 5.8 and 6.7, being stronger and more uniform at pH 6.7 in the presence of high concentrations of LF (20% and 30%). Those findings revealed both the practical and theoretical significance for the utilisation of LF in gelled protein products.

Theoretical models and numerical simulations were applied by Delboni and da Silva [25] to gain insight into understanding the fundamental mechanisms responsible for the process of milk protein complexation under different conditions. This knowledge can be essential to use milk proteins in nanoscale encapsulation systems for food and pharmaceutical applications. The interactions between β-Lg and LF and between α-La and LF were investigated by means of Monte Carlo simulations. The comparison between the free energies associated with the complexation of LF with β-Lg and α-La at different pH and ionic strengths revealed the weaker attraction between α-La and LF in contrast with the stronger attraction measured between β-Lg and LF. The driving force for the complexation being studied of β-Lg with LF is due to an association of electrostatic interactions, where protein charge plays an important role.

The influence of small ligands on the complex coacervation between β-Lg and LF was studied by Tavares et al. [32]. In this work, 8-anilinonaphthalene-1-sulfonic acid (ANS), a fluorescent probe, was used as a model of a small ligand. While ANS did not interact with β-Lg, it presented two binding sites for LF inducing its self-aggregation. Depending on its concentration, ANS modulated the shape of the β-Lg-LF macromolecular assembly. Coacervates were observed for ANS/LF molar ratio <25 against amorphous aggregates found for higher ANS/LF molar ratios.

In 2020, Darmawan et al. [33] explored a novel in silico approach to study the possibility of employing whey protein components to encapsulate LF. The interaction of apo-LF (the most unstable form of the protein) with β-Lg and α-La was studied at temperature conditions that are used during the spray drying process (95 and 180 °C). This method is commonly employed in the food industry to encapsulate food ingredients by using hot air drying. It was found that the interaction of β-Lg and α-La with LF allowed maintaining the structure of two regions of the molecule corresponding to antibacterial peptides (lactoferricin and lactoferrampin) under the spray drying conditions. In this study, it was also demonstrated that apo-LF was most probably readily dispersible during subsequent rehydration due to its tendency to agglomerate under those temperatures. Moreover, they showed that the interactions between apo-LF, β-Lg and α-La were due to the acidic and basic amino acid residues of these proteins.

In the study by De Figueiredo Furtado et al. [34], LF was proposed as a promising ingredient for incorporation into powder infant formulas, because it improved their properties compared with formulas without this protein. The interaction of LF with WPI or whey protein hydrolysates used in the formulas, as encapsulating components of the oil mixture added, resulted in more stable emulsions. The powders obtained from the emulsions containing LF were those with a lower stickiness and consequently, with higher yield, encapsulation efficiency and wettability.

- Caseins

Caseins represent the major protein fraction of milk, making up 80% of the total proteins. They precipitate at pH 4.6 and individual caseins, including αs1, αs2, β and κ-casein, are unique proteins with respect to their structure and function. They have open and flexible conformations and consist of hydrophilic and hydrophobic segments. Due to their highly hydrophobic nature, individual caseins are stabilised by the creation of a micellar structure, which is chemically heterogeneous and is composed of the four types of caseins and by amorphous calcium phosphate [35]. The stability of the caseins and casein micelles to some treatments, such as heating, freezing, and drying, make them valuable in delivering food ingredients and bioactive compounds [36].

Anema and de Kruif investigated the interaction of LF with the casein micelles, as found in bovine skimmed milk [37], and with the individual caseins [38]. The cationic LF (pI 8.3–8.7) was found to bind to the anionic caseins (pI range from 4.9 to 5.6) and to the casein micelles (pI 4.6) at intermediate pH values. The binding of LF to the individual caseins can be described as the formation of complex coacervates. Moreover, Anema and de Kruif [39] assayed the binding of LF to transglutaminase (TGA) cross-linked casein micelles. They found that the internal cross-linking of the casein micelles by TGA did not alter the binding mechanism of LF to them. The binding of LF to the untreated micelles swelled the micelles, which was avoided in the TGA-micelles. Additionally, after several hours in the presence of added LF, the untreated micelles started to disintegrate, and their size decreased (increasing transparency of the milk was observed). In contrast, the internal cross-linking of the casein micelles prevented the disintegration of the casein micelles.

In 2016, Anema and de Kruif [40] studied complex formation between some bovine casein types (α-casein, ACN; β-casein, BCN, and κ-casein, KCN) and LF. They showed that the optimum coacervation occurred at the mixing ratio in which charge neutrality happened and the turbidity was maximum. The kinetics of complex formation for LF/BCN and LF/KCN were rapid and occurred through a nucleation and coalescence process. However, the kinetics of complex formation for LF/ACN were much slower. They evidenced that when salt is added the coacervation diminishes, leading eventually to a one-phase system. Salt decreases the entropical contribution, and consequently, the coacervation is not observed. For the LF/BCN or LF/KCN coacervates, the samples were completely transparent (turbidity of ~0) at an ionic strength of ~25 mM, whereas for the LF/ACN, a much lower ionic strength of only ~10 mM was required for the sample to be completely transparent. It is interesting to read the review article of Anema [41], in which he summarises all the results that have been obtained in his research group studying the spontaneous self-association of LF with the casein micelles in milk and with individual isolated caseins.

The complexation behaviour between LF and sodium caseinate (NaCas) before and after heat treatment was also studied by Li and Zhao [42]. The results show that the denaturation and aggregation of LF can be inhibited by forming soluble LF-NaCas complexes. The complexes formed at a ratio of 2:1 had an average diameter of 194 nm and exhibited a higher capacity for lowering the air/water interfacial tension compared to complexes with a lower proportion of LF. NaCas played a role as a stabiliser to protect LF against heat-induced precipitation. Therefore, the presence of NaCas is important for maintaining the structure and functionalities of LF during the production of LF-enriched food products. However, the authors have not demonstrated the biological activity of LF while being part of the complexes formed.

#### 3.1.2. Encapsulation of Lactoferrin with Other Proteins

The ability of LF to form positively charged droplets at neutral pH can have many important practical implications. Recently, there has been an increasing interest in the application of whey proteins, and especially LF, as emulsifiers [43]. LF has been used as an emulsifier in multilayer emulsions for lipophilic nutraceutical delivery of active compounds, such as curcumin and β-carotene [44]. LF can interact with other proteins or polysaccharides, providing good stability to oil-in-water emulsions [45].

**Figure 3:**
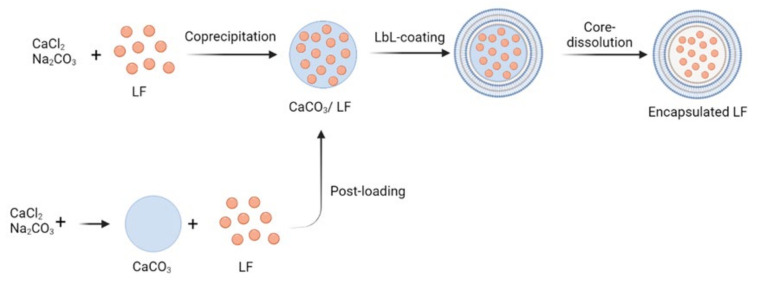
Scheme of LF encapsulation, co-precipitation of CaCO_3_ and LF vs. post-loading of LF in porous CaCo_3_ microparticles, both followed by Layer-by-Layer (LbL) deposition of bovine serum albumin and tannic acid and final dissolution of CaCO_3_ (Redrawn and slightly changed from that contained in the article by Kilic et al. [46], which is an open access article distributed under the terms and conditions of the Creative Commons Attribution (CC BY) license (http://creativecommons.org/licenses/by/4.0/, accessed on 30 October 2021).

BSA with tannic acid (TA) has been used to encapsulate LF for oral administration by alternating layers of BSA and TA on porous microparticles of CaCO_3_ previously prepared with LF [46]. The authors examined two approaches to load LF into CaCO_3_ particles, as shown in Figure 3. The first one used co-precipitation in which CaCO_3_ microparticles were directly formed in an LF solution. The second one used post-loading in which CaCO_3_ microparticles were prepared in a water solution and then dispersed in an LF solution. The CaCO_3_ was dissolved after covering the microparticles with the layers of BSA and TA. The post-loading approach showed lower LF degradation than that of co-precipitation and was chosen as the oral delivery system in a murine model. The microcapsule shells formed conferred LF high stability and protection in gastric conditions, while they were degraded under intestinal conditions, thus releasing LF. The animals dosed with encapsulated LF showed a 6.5 times higher concentration of LF in the intestine than the control group dosed with free LF. Moreover, LF was later detectable in the liver, demonstrating that this encapsulation system has great potential for oral delivery of bioactive molecules.

Pea protein isolate (PPI) has also been used to form complexes and coacervates with LF by electrostatic interactions under specific pH conditions [47]. The maximum level of coacervate formation was observed at charge neutrality. The pH where coacervation was most favourable was pH 5.4, and the soluble complexes were maximised at pH 7. The formation of heteroprotein complexes was studied by Small Angle X-ray Scattering (SAXS), confirming the formation of complexes of around 13 nm at pH 7 and around 80 nm at pH 5.4. A predominance of elliptical over spherical shapes for LF-PPI coacervates was found, and rare chain-like aggregations were observed, probably due to PPI-PPI complexes. These coacervates can be of interest in various food applications.

Zheng et al. [21] investigated the conditions, thermodynamic mechanism, and morphological structure for the formation of soy protein isolate/lactoferrin (SPI/LF) complex coacervate. The stoichiometry of two optimal complex coacervates (SPI/LF = 1:3 at pH 6.25 and SPI/LF = 1:4 at pH 6.6) was identical to their mixing ratio. Moreover, the SPI/LF complex coacervation was exothermic and accompanied by significant entropy gain. This coacervation was established by electrostatic interactions and by hydrogen bonds. It was shown that the SPI/LF interaction improved the heat-stability of the LF heat-sensitive lobe. When the structure of the complexes was analysed, it was observed that the 1:3 SPI/LF complex exhibited distinct granules, whereas the 1:4 SPI/LF complex presented a uniform cross-linking structure. Analysis by atomic force microscopy showed the existence of sphere complexes with a diameter of 50–150 nm in the 1:3 SPI/LF complex and a chain-like structure with a length of 50–150 nm and a width of 20–80 nm in the 1:4 SPI/LF one. This different structure was hypothesised to be by SPI self-aggregation. It remains unanswered if LF maintains its properties in SPI/LF coacervates, although the increase observed in LF thermal resistance is very positive for the potential applications of those coacervates.

#### 3.1.3. Encapsulation of Lactoferrin with Polysaccharides

Biopolymer nanoparticles have gained popularity thanks to their exceptional physical characteristics, such as biodegradability, biocompatibility, low toxicity and great ability to bind hydrophobic bioactive compounds [48,49]. The protein-ionic polysaccharide electrostatic attraction requires a specific pH, in which the charges of these molecules are opposite. The net charge of a protein at its pI is zero, being positive below the pI, and negative at a pH higher than the pI. Thus, cationic and anionic polysaccharides can form electrostatic complexes with proteins depending on pH. At physiological pH, LF is positively charged (pI 8.3–8.7); therefore, its capacity to create complexes with several anionic polysaccharides has made possible the extensive research produced in this area [49].

The type and concentration of protein and polysaccharide, as well as the temperature, pH and ionic composition of the solution, determine the functional characteristics of a protein-polysaccharide complex [50]. Additionally, the formation of complex coacervates is influenced by the molecular weight, charge density and chemical nature of the polymers used [51].

- Alginate

Alginate is a natural, biodegradable and biocompatible anionic polysaccharide obtained from brown algae [52]. It is an organic polymer derived from alginic acid and is located in the cell wall of the algae. Its structure consists of linear chains composed of monosaccharides D-mannuronic and L- guluronic.

This polymer owes its polyanionic character to the carboxyl groups that appear along the chain. The distribution of monomers in the polymeric chain and the charge and volume of carboxyl groups give to the formed gel characteristics of flexibility or rigidity depending on the guluronic content. The higher the number of guluronic blocks, the harder and more rigid and brittle the gel is, while if the mannuronic groups predominate, the gel is softer and more elastic [53]. This polymer is presented as sodium alginate (Na-Alg) when used as a food additive and is easily available and widely applied in the industry. The main characteristics of Na-Alg are its good affinity with water and its highly anionic charge at pH values higher than two [54].

On the other side, alginate combined with calcium (Ca-Alg) forms a gel that is a well-known system used for the elaboration of gel beads. These beads are useful to encapsulate a wide variety of bioactive agents, due to their simplicity, biocompatibility, non-toxicity and low cost [5].

When two chains of the guluronic blocks align, coordination sites are created. These chains are in the shape of loops, creating cavities with adequate size to accommodate the calcium ion. After the addition of calcium, the alginate undergoes conformational changes, producing the well-known “egg box” gelling pattern (Figure 1 in [53]). This pattern is based on the dimerization of the chain and, later on, further aggregation of the dimers [53].

Furthermore, alginate is a good option for the encapsulation of molecules since it has unique characteristics that allow a controlled release in the intestine [6]. Additionally, alginate microbeads, reaching a mean diameter of 130 ± 47 µm, are one of the most studied encapsulation systems [55]. Braim et al. [6] and Kanwar et al. [56] confirmed that the LF-alginate capsule can be coated with chitosan, another polycationic polymer, to improve the integrity of the capsule in GIT fluids.

Some studies have proven the efficacy of coating LF by alginate capsules to deliver this protein to the colon [56] and to impede the growth of Clostridiodes [6]. Furthermore, the use of LF as a complement to alginate to encapsulate essential oil particles [52] or probiotics [55] has also been achieved.

In the study by Raei et al. [5], LF was encapsulated in Ca-Alg capsules, and it was proven that the effectiveness of these capsules increased with higher concentrations of Ca-Alg and with thermal treatment (61 °C for 10 min). However, the stability of the nanoparticles was greater when the polysaccharide concentration was lower (0.2%, *w*/*w*) and the thermal treatment was applied. These particles remained intact for 30 min at pH 2, conditions that can be considered equivalent to gastric digestion, which guarantees a controlled release during the first 30 min and a subsequent gradual release in acidic and neutral conditions. In this way, they confirmed that the encapsulation of LF with Ca-Alg is a technique that can be used for the targeted delivery of LF to the intestine.

Furthermore, Bokkhim et al. [57] encapsulated three different forms of LF (apo-, native- and holo-) in micro-gel particles of alginate, using the aerosol technique. They produced the particles from a 2% (*w*/*w*) solution of LF/alginate (1:1), using 0.1 M CaCl_2_ as the cross-linking solution. These authors demonstrated that a major concentration of CaCl_2_ decreased the encapsulation efficiency. LF/alginate particles were maintained intact- during simulated gastric digestion of 2 h, with 30% more protein compared to the non-encapsulated LF. The digestion of all forms of LF, pure or encapsulated, under intestinal fluids was rapid. Thus, these authors showed with this study that LF/alginate micro-gel particles can protect LF from the action of pepsin (the main enzyme of gastric digestion) and release it at the intestine.

Additionally, Wang et al. [54] proved the efficacy of the LF/Na-Alg complex coacervates. This study showed that owing to the positive charge of LF at pHs lower than 8.5 and the negative charge of Na-Alg in the entire pH range studied (2–10), the electrostatic interaction between both molecules was excellent. This electrostatic interaction increased as the pH decreased from eight to five and remained constant at pH from five to four. Therefore, the authors concluded that the optimum pH for the formation of the LF/Na-Alg complex coacervates was 4.5. Additionally, they compared the degradation of the uncoated LF and that of the alginate capsule in the gastric stage. They found that 100% of the uncoated protein was degraded under gastric conditions. This percentage decreased to 70% when LF was protected with Na-Alg, keeping 30% of the protein intact at that stage of digestion. Alginate provided a blockage of the sites of LF for the action of pepsin, reducing LF proteolysis. Several authors have defended that alginate can interact with pepsin, decreasing its catalytic mechanism during gastric digestion, while it does not have the same effect with trypsin, being degraded by this enzyme in the intestinal digestion [52,54,57].

The encapsulation of LF with Na-Alg allows the preservation of its properties, such as its antioxidant activity, increasing by 30% with respect to the uncoated LF under gastric conditions (pH 2). After gastric digestion, the antioxidant capacity of uncoated LF decreased by 12% due to the breakdown of peptides. However, the antioxidant capacity of LF/Na-Alg coacervates was not modified after this digestion stage [54].

- Chondroitin sulphate

Chondroitin sulphate (ChS) is a polyanionic mucopolysaccharide that is used as a food-grade material and is obtained from animal cartilage. ChS contains strongly acidic sulphate groups and weakly acidic carbonyl groups, so it has a high negative charge density and can form ionic complexes with positively charged molecules, such as LF. Furthermore, the ability of ChS as a potential carrier for drug delivery has been investigated, as it possesses numerous attractive properties, such as biosafety, biocompatibility and biodegradability [48]. Additionally, ChS is considered a possible ligand targeting cancer cells, which overexpress CD44 receptors [58] and it is valued as a suitable polysaccharide for delivering active compounds to target cells [48].

In the same study by Abdelaziz et al. [58], it was proposed to coat the surface of nanoparticles with ChS and LF through carbodiimide coupling or by electrostatic interactions, as a potential approach to attack cancer cells overexpressing LF receptors. Here, the use of LF and ChS as coating materials demonstrated their efficiency in tumour selection.

- Galactomannans

Galactomannans are industrial polysaccharides obtained from seeds of the Leguminosae family. They are storage polysaccharides formed by a skeleton of mannose with galactose branches. They are used to increase viscosity or film production. Galactomannans have applications in both food and clinical industries, due to their rheological properties [59]. In increasing the order of mannose:galactose ratio, we can distinguish different types of galactomannans: fenugreek gum (1:1), guar gum (2:1), tara gum (3:1) and locust bean gum (4:1) [60].

Nanoparticles containing LF have been formed from a soluble polyelectrolyte complex composed by LF/N-succinyl chitosan/galactomannan in a 1:3:2 ratio, after heat treatment below the protein heat denaturation temperature. The presence of galactomannan allows forming the particles, not only by electrostatic interaction but also by hydrophobic interactions and hydrogen bonds. Because of those interactions, LF is well integrated into these complexes, due to the hydrophobic interaction between the pyranose chains of chitosan, galactomannan and the hexose residues of the hydrocarbon component of LF [61]. These biopolymer particles have a 250–400 nm size, regular spherical shape and good storage stability. The load of LF in the particles was 76–87% and they retained their characteristics after lyophilisation. Therefore, these particles can eventually be used for the targeted transport of biologically active substances [49].

The immobilization of LF in galactomannan films was proposed by Albuquerque et al. [59] as an alternative to conventional edible solid dosages, such as capsules, particles, beads or tablets. This alternative can be effective for biotechnological applications in the pharmaceutical and the food industries, for individuals with dermal wounds or with difficulty in swallowing edible doses, for example.

The tara gum, like the rest of galactomannans, is a neutral polysaccharide; therefore, the insertion of charged groups into its structure allows its interaction with other polymers and proteins. The process of carboxymethylation consists of the insertion of negatively charged carboxymethyl groups in the main polysaccharide, thus favouring the formation of complex coacervates [62]. Taking the advantage of this property, Santos et al. [63] performed the microencapsulation of vitamin D3 through complex coacervation in matrices formed by carboxymethyl tara gum and LF. To promote the electrostatic interaction between tara gum and LF, the system was shaken for 10 min at 300 rpm and then quickly placed in an ice bath to reduce the temperature. The addition of TGA to these structures catalysed the formation of covalent bonds between the proteins, increasing the stability of the particles formed. It was verified that a ratio of 1:3 (core:wall) was enough for correct encapsulation of the vitamin to ensure that it was protected by the polymeric matrix.

The complex coacervation of LF with biopolymers increases its stability during gastric digestion since the acidic pH of the stomach maintains both polymers with opposite charges supporting their interaction. However, intestinal pH (7) has a negative effect on coacervation since this pH modifies the positive charge of LF necessary for the interaction with tara gum [63]. Therefore, these complexes improved the stability and facilitated the controlled release of the vitamin during GIT digestion.

- Gum arabic

Gum arabic (GA) is an anionic polyelectrolyte that mainly consists of three fractions of anionic branched arabinogalactans. These fractions are 80–90% arabinogalactan, 10–20% arabinogalactan-protein and 2–4% glycoprotein. The emulsifying properties of GA are mainly attributed to the arabinogalactan-protein [50]. Furthermore, due to its low viscosity and high solubility at high concentrations, and its emulsifying and encapsulating capacities, GA is extensively used in industry [51].

The protein-polysaccharide electrostatic complexes can be used as stabilisers and emulsifiers in high internal phase emulsions (HIPEs). Some authors determined that the LF/GA complexes (1:1) can be used to form HIPEs over an ionic strength range of 0–300 mM NaCl at a pH range of 3–6. During the homogenisation applied for this process, GA is not as efficient as proteins at producing small droplets; but it is a better stabiliser once the droplets have been formed as reported by Cheng et al. [50]. These authors also showed that the antioxidant properties of LF allowed, in these HIPEs of LF/GA complexes, the encapsulation of nutraceutical compounds, such as curcumin, protecting them from chemical degradation.

- Pectin

Pectin is an anionic polysaccharide present in plants, mainly in fruits and young tissues. It is made up of galacturonic acid units with free carboxyl groups, which give it an acid tendency. In its native form, pectin is highly methylated. The degree of pectin methylation influences its ability to form gels. This polysaccharide is easily degradable under alkaline conditions and, especially, at high temperatures [64]. Pectin is a polysaccharide most used to produce multilayer encapsulation systems with whey proteins, and in particular, it has allowed the encapsulation of LF at pH between three and four with a size of around 700 nm and good characteristics to be used in pharmaceuticals and food products [65]. In another study by Bengoechea et al. [64], electrostatic complexes between high-methoxyl pectin and LF were formed at pH 7. These complexes were soluble at pH between 7 and 3.5 but underwent aggregation at lower pH. The properties of these pectin/LF complexes can be modulated through the pH, temperature or biopolymer concentration. Furthermore, it was observed that at pH 7 the stability of LF/pectin complexes to aggregation during heating was much better than that of LF alone.

Niu et al. [66] also created pectin/LF complexes by mixing the two biopolymers in solution, leading to spontaneous colloid formation due to electrostatic interactions. They compared the potential of low (LM) and highly methylated (HM) pectin to interact with LF and determined the loading of LF in the capsules and their encapsulation efficiency. They concluded that LM pectin had a greater capacity to encapsulate LF, with an encapsulation efficiency of 40%, compared to 5% for HM pectin. Additionally, they verified the inhibitory effect on bacterial growth of the LF-pectin complexes at a concentration of 1 mg/mL, an effect that lasted at least 48 h. Therefore, they stated that LF encapsulation with pectin did not affect its bioactivity.

Moreover, in the study conducted by Niu et al. [66], some pectin-LF complexes were coated with chitosan in order to increase their gastric stability and muco-adhesive properties. This coating improved the colloidal stability of the complex in simulated gastric fluid. Furthermore, in an HCl solution at pH 3, both systems, the uncoated and the chitosan-coated LF/pectin complexes, showed a gradual release of LF. After 2 h under these conditions, the uncoated complex showed a 30% release of LF, while the chitosan-coated complex only released 10%. However, under gastric simulated conditions (pH 3, no pepsin), the uncoated complex showed a negligible release of LF. This limitation of LF release indicates that this protein would not be as exposed to digestive enzymes, such as pepsin. Therefore, these pectin/LF systems can be a good way to incorporate LF in foods or special products for oral administration, as they decrease the hydrolytic effect of pepsin on LF, protecting this bioactive molecule from gastric digestion up to at least 60 min for a gut-targeted delivery [66].

- Chitosan

Chitosan is a useful natural biopolymer, obtained by chemical or enzymatic deacetylation of chitin, a polysaccharide present in nature (insects, crustaceans and fungi) [67]. It has multiple reactive groups that can help in the transport of drugs due to its physicochemical properties, such as charge and hydrophobicity. Thus, due to these reactive groups, chitosan nanoparticles can be complemented with targeting vectors like LF [68]. These authors showed that LF and succinyl-chitosan formed stable and high-yield nanoparticles and that the globular structure of LF allowed the formation of stable nanoparticles without chemical conjugation. These nanoparticles were designed to bind to immune cells, such as macrophages or phagocytic cells, in a few minutes and deliver LF by cross-presentation, the mechanism of antigen-presenting cells (APCs). Thus, the encapsulation of LF into chitosan can change the traffic of proteins inside APCs and modify the immune responses.

LF was found to form nanohydrogels by thermal gelation with the glycomacropeptide (GMP) showing high encapsulation efficiencies for curcumin and caffeine and a controlled release of those compounds [69]. Later, the same authors [70] studied the application of chitosan coating in LF-GMP nanohydrogels. This biopolymer did not affect the shape of nanohydrogels, maintaining their spherical shape and decreasing their aggregation in the solution. These authors evaluated the effect of chitosan-coat on release mechanisms of nanohydrogels at gastric digestion, using caffeine as the encapsulated compound. They concluded that the presence of chitosan improved the stability of LF-GMP nanohydrogels, keeping them intact until 60 min under acidic conditions, compared with the 15 min that the uncoated nanohydrogels remained intact. They continued their study and, in 2018, revealed that chitosan coating reduced the protein degradation from 86% to 76% for LF and from 71% to 53% for GMP. Furthermore, the chitosan increased the bioaccessibility of curcumin (bioactive compound encapsulated in the nanohydrogel) and protected its antioxidant activity during the GIT digestion, losing only 30% of it compared to the 68% of activity loss for free curcumin [71].

Throughout this review, it has been shown that chitosan can be combined with other polysaccharides to favour the stability and integrity of the formed particles [6,49,61,66]. As an example, chitosan together with pectin has been used to stabilise LF-loaded liposomes obtaining solid particles that are more resistant than liposomes to heat and to simulated intestinal digestion [72].

### 3.2. Nanocarriers Coated with Lactoferrin

The advances in the design of targeted drug delivery systems have been very relevant in the last few decades. These delivery systems must consider the surface properties and receptors of the target cell, the properties of the carrier and the suitable ligand to be utilised. Moreover, a targeted drug delivery system should be non-immunogenic, biocompatible, specific to the target site, and show chemical and physical stability, both in vitro and in vivo [73].

There are two strategies to design targeted delivery: passive and active. In the passive strategy, drugs are loaded into nanocarriers of a size around 200 nm, which can reach the tissues or organs by their enhanced permeability and retention. In the active strategy, drug-loaded nanocarriers are conjugated with a certain ligand, which can further bind to a specific receptor on the surface of target cells. This receptor is normally overexpressed in the affected cells by the disease and consequently, the drug accumulates on them [73].

Receptors for LF have been expressed in many cells, such as brain endothelial cells, liver cells, epithelial respiratory cells, cancer cells, etc. Therefore, LF can be used to cover nanocarriers loaded with drugs to increase their delivery to the cells via LF interaction with its receptors. Furthermore, the cationic nature of LF allows its binding to anionic cellular compounds, such as glycosaminoglycans [73].

In the last few years, inorganic nanoparticles have attracted considerable attention for drug delivery and imaging applications due to their characteristics. The positive features of these nanoparticles are their higher loading capacity, ease of functionalization with several types of ligands, biocompatibility and adaptable degradation rates. There are several types of nanocarriers coated with LF that have been developed for delivery applications.

Thus, superparamagnetic iron oxide nanocarriers (Fe_3_O_4_-LF) with narrow size ranges were developed via surface functionalization with LF using the EDC (1-ethyl-3-(3-dimethylaminopropyl) carbodiimide hydrochloride) coupling reaction aiming to target the receptors that are highly expressed on the surface of human fibroblasts [74].

Superparamagnetic iron oxide nanoparticles conjugated with LF (LF-SPIONs) [75] and LF-PEG-Fe_3_O_4_ [76] were successfully fabricated via the EDC coupling reaction and were applied as a magnetic resonance imaging contrast agent for improving the detection of in vivo brain glioma.

Many other applications of superparamagnetic nanocarriers coated with LF have been applied in antitumour treatments [77,78,79]. LF has been covalently attached to silica nanoparticles by EDC coupling reaction and then PEGylated to extend the blood circulation time [73]. The LF coated silica nanoparticles have been revealed as effective against breast cancer and as able to cross the brain–blood barrier [78,80]. There are many applications of nanoparticles and nanocarriers functionalised with LF to treat different pathologies, and they have been compiled in Table 1.

### 3.3. Edible and Active Film Composites with Lactoferrin

The processing of edible coatings and films is based on the use of various compounds with different properties, such as polysaccharides, proteins and hydrocolloids. A method to improve the properties of edible films is mixing polysaccharides and proteins, which is a promising area of research [140].

The development of new edible films has a great interest in the industry and for consumers, who are concerned about the need to reduce the use of plastic packaging for environmental reasons [141]. Several compounds have been used to develop edible films, trying to preserve the sensorial properties and safety of foods. On the one hand, the use of edible films containing antimicrobial agents has certain advantages over their direct application on the food surface, as they can be designed to control the speed of antimicrobial diffusion to the surface of food [142]. There are several techniques used to incorporate antimicrobial agents into packaging films. Some techniques are inappropriate for sensitive compounds, like those involving direct incorporation in the polymer matrix, whereas other strategies like film coating are much more adequate [143].

Chitosan is a natural polymer that is biodegradable, biocompatible and presents antimicrobial properties. LF has been incorporated into chitosan films to improve barrier properties and to enhance the preservation of foods due to its antibacterial activity [144]. In the study by Brown et al. [145], a chitosan film containing LF alone was not found to be effective against *Listeria monocytogenes* and *E. coli* O157:H7, though when it was combined with lysozyme (LYS), the antibacterial activity of LYS was increased more than when it was combined with EDTA. A mixture of LF and LYS was also incorporated into cellulose-based packaging material and was proved to be effective against some meat contaminants, such as *Escherichia* and *Listeria*, also limiting the increase of natural microbiota present in veal meat [146].

Cellulose films from bacterial origin containing LF have been proven to be non-toxic and have adequate technological characteristics to be used as bio-based meat product casings, showing a bactericidal activity against *E. coli* and *Staphylococcus aureus* [147]. The methodology used for binding LF to cellulose films has a great influence on its antibacterial activity. Thus, Padrao et al. [148] compared the binding of LF to bacterial nanocellulose (BNC) by embedding the protein within the three-dimensional structure of BNC with its covalent binding to BNC nanofibrils. The concentration of LF in BNC obtained using the first method was twice that obtained with the second one, but LF only maintained a significant bactericidal activity against *E. coli* and *S. aureus* in the first type of BNC.

Starch is one of the most widely used materials within the bioplastic industry, due to its biodegradability, renewability, availability and low cost [149]. Starch does not have thermoplastic properties, though, with the aid of additives, it gels producing thermoplastic starch (TPS) that has been used for developing films. Thus, in the study by Moreno et al. [149] LF and LYS were incorporated into the starch film, providing it with beneficial properties. Neither LF nor LYS was effective enough as antimicrobials when they were applied in the film separately, though the combination of both proteins weakly enhanced their antimicrobial activity against *E. coli* and coliform microbiota of pork-minced meat. The films containing a blend of LF and LYS also reduced lard oxidation after long storage times [149].

In the study by Tavassoli et al. [150], multifunctional films were created by embedding different kinds of functional nanoparticles into a gelatin-based film prepared using a casting method. The nanoparticles were prepared by cross-linking cationic chitosan nanofibers, and afterwards, quercetin, LF, or both were introduced into these nanoparticles. The incorporation of the nanoparticles into the films decreased their mechanical strength and stiffness but increased their flexibility. The presence of LF, quercetin and chitosan in the gelatin-based films increased their antimicrobial and antioxidant activity. Furthermore, the incorporation of the nanoparticles into the films improved their degradation under simulated environmental conditions.

Bovine LF and its derived peptide lactoferricin B were individually immobilised on two different coatings. The coatings were functionalized with carboxyl groups deposited in the inner part of polyethylene microtubes by using a plasma deposition process fed with ethylene and acrylic acid vapours [151]. The resulting functionalized tubes were tested for antimicrobial activity against three *Pseudomonas* strains responsible for casein hydrolysis and cheese pigmentation in Mozzarella. It demonstrated the antibacterial activity of immobilized lactoferricin B, with a significant reduction in the growth of the bacteria tested, though no activity was observed for the immobilised LF. Many studies have been conducted showing the incorporation of LF, alone or combined with other molecules, in active and edible films, which are compiled in Table 2.

## 4. Discussion

The current review has evaluated the encapsulation strategies to protect LF and its bioactivity. These techniques have been developed in recent years to preserve bioactive compounds incorporated into pharmaceutical, food and cosmetic products.

Nanoencapsulation is defined as a process in which very small particles are surrounded by a coating or embedded in homogeneous or heterogeneous matrices. This process can improve the bioavailability and solubility of bioactive compounds, enhance their residence time and stability in the GIT and give them a better ability to enter and permeate tissues and cells.

During the last few years, many studies have detailed encapsulation by combining a protein, specifically LF, with milk proteins, other proteins or polysaccharides of different origins. Furthermore, numerous articles focus on nanocarriers coated with LF or edible and active film composites with LF.

Many studies have demonstrated the interaction of LF with proteins of different origins, including milk proteins (such as β-Lg, α-La and caseins), pea or soy proteins. The main interaction mode is by means of electrostatic interactions, based on the basic nature of LF. However, the biological activity of LF in these systems has not been demonstrated in many cases. The binding of LF to whey proteins, caseins or soy proteins increases LF thermal resistance, which shows great potential for different applications in industry.

LF can be useful for the delivery of different molecules. In that sense, it has been shown that LF, by forming coacervates with β-Lg, can act as a carrier for the release of vitamin B9. LF can also be used as an emulsifier for the release of other active compounds, such as curcumin or β-carotene and forms more stable emulsions in infant formulas.

Only one system based on proteins has been identified for the oral delivery of LF itself. BSA, with tannic acid (TA), has been used to encapsulate LF for oral administration by alternating layers of BSA and TA on porous microparticles of CaCO_3_, previously prepared with LF. This system has been studied in an animal model showing promising results, as LF was identified in the liver, demonstrating that it can pass the GI tract.

The formation of the protein-polysaccharide complex depends on various factors, such as pH, molecular weight and charge density. There are numerous polysaccharides that can be combined with LF: natural like alginate or chitosan, or industrial like galactomannans. In any case, all of them allow the encapsulation of LF or the combination with this protein to transport bioactive compounds such as vitamin D3, curcumin or nutraceutical compounds, protecting their bioactivity and integrity against chemical degradation.

Many studies have focused on the formation of these complexes by electrostatic interactions, taking advantage of the positive charge of LF and the opposite charge of the polysaccharides, such as alginate or chondroitin sulphate. This complex coacervation of LF with biopolymers increases its stability during gastric digestion since the acidic pH of the stomach maintains both polymers with opposite charges supporting their interaction. However, intestinal pH has a negative effect on coacervation, modifying the positive charge of LF necessary for the interaction with the polysaccharide. Therefore, these complexes improved the stability and facilitated the controlled release of the protein during GIT digestion. Furthermore, galactomannans allow, in addition to electrostatic interaction, the formation of films by hydrogen bonds or by hydrophobic interactions that favour a good integration of LF.

In addition to a controlled release in the gut, some polysaccharides allow delivery to target cells. While chondroitin sulphate combined with LF targets cancer cells, being effective for tumour selection, chitosan-LF nanoparticles bind immune cells and deliver LF by cross-presentation to macrophages or phagocytic cells. Several authors have used chitosan to coat LF-polysaccharide complexes, increasing the integrity of the capsule and its stability under gastric conditions.

The strategy of coating nanoparticles with LF has been developed intensively over the past few years. The receptors for LF are highly expressed on the surface of many cells; therefore, nanocarriers functionalised with LF have been used to treat tumours of several origins, such as mammary, hepatic, pancreatic and colonic, among others, with different results. However, there are still some issues to be investigated to know the interaction of LF-nanocarriers with the capillary endothelial cells in the blood–brain barrier and the kinetics of LF interaction with the surface of different types of cells. The use of LF nanocarriers to enhance the imaging analysis of some brain tumours is also an area of great interest for future development.

The food industry is currently investing in the development of edible and biodegradable films with properties directed to improve food preservation and to avoid the use of plastic packaging. Furthermore, the incorporation of bioactive molecules into the films has been encouraged over the past few years. These new films are able to maintain food quality and safety and, at the same time, allow reducing the use of chemical additives. In many studies, LF has been used in active films because of its antimicrobial properties. However, the results have indicated the existence of some problems in maintaining LF activity in the process of combining it with the base materials to build films or packaging. Some studies have proved that the combination of several active molecules with films, such as LF and LYS, gives better results. Nevertheless, the enrichment of films with bioactive molecules adds a high cost to the final product, a limitation that should be resolved to make those films applicable to food industry.

## 5. Conclusions

Lactoferrin is a molecule with many biological properties that must be preserved when it is used in products to improve health or is incorporated into active films designed to maintain the quality of food products. The encapsulation of this bioactive protein with other proteins or polysaccharides prevents its proteolysis during gastric digestion, allowing a targeted release of LF. This protein can also be used in the active targeted drug delivery, where drug-loaded nanocarriers are covered with LF, which can further bind to its receptors expressed on the surface of target cells. Future research on all these issues should be continued by evaluating the different LF activities, after being encapsulated or combined with certain composite materials, in in vitro assays and eventually, in humans.

## Figures and Tables

**Table 1:** Studies regarding the use of lactoferrin in different nanocarriers for therapeutic applications.

Application	Type of Nanocarrier	Model of Study
**Cancer therapy**		
Brain tumour	Magnetic nanocarriers	Rats [77]
	Solid lipid nanoparticle	HBMECs, U87MG cells [81]
Breast cancer	Polymeric nanoparticles	MDA-MB-231 cells, mice [82]
	Silk nanoparticles	MDA-MB-231, MCF-7 cells [83]
	Protein nanocapsules	Mice [84]
	Quantum dots-based chondroitin sulphate nanocapsules	MCF-7, MDA-MB-231 cells, mice [85]
	Zein nanospheres	MCF-7, 4T1 cellsrats [86]
	Polymeric micelles	MDA-MB-231, MCF-7 cells [87,88]
	Silica nanoparticles	MCF-7 cells [80]
	Betulinic acid nanoparticles	MDA-MB-231 cells [89]
	Magnetic nanoparticles	4T1 cells [79]
Bronchogenic carcinoma	Solid lipid nanoparticles	BEAS-2B cells, rats [90]
Colon tumour imaging	Polymeric nanocarriers	Caco-2 cells, mice [56,91]
	Polymeric nanocarriers	Caco-2 cells, mice [92]
	Protein nanoparticles	COLO-205 cells, rats [93]
Glioblastoma	Polymeric nanoparticles	BCEC, C6 glioma cells, rats, mice [94]
	Polymeric nanoparticles	U87MG, HBMEC, HA cells [95]
	Protein nanoparticles	U87MG, BCEC, HUVEC cells, mice [96]
	Protein, FePt nanoparticles	U87MG, U-373 MG, MDCKII cells, rats [97,98]
	Gadolinium oxide nanoparticles	U87MG, MCF-7 cells, mice [99]
Glioma	Magnetic nanoparticles	HEK 293, C6 glioma, ECV 304 cells, rats [75]
	Magnetic nanogels	C6 glioma, ECV 304 Rats [100]
	Protein nanoparticles	BCECs, C6 glioma cells, rats [101]
	Magnetic nanoparticles	C6 glioma cells [102]
	Magnetic nanoparticles	C6 glioma cells, rats [103]
	Polymeric nanoparticles	C6 glioma cells, rats [104]
	Magnetic nanocomposites	C6 glioma cells [78]
	Polymeric nanoparticles	BCECs, C6 glioma cells, mice [105]
	Polymeric nanoparticles	L929, glioma 261 cells [106]
Hepatocellular carcinoma	PEGylated liposomes	HepG2, ECV304, BEL7402, NIH 3T3, SMMC7721 cells, mice [107,108]
	PEGylated liposomes	HepG2 cells [109]
	Protein nanoparticles, nanoemulsion	HepG2 cells, mice [110,111]
Laryngeal cancer	Betulinic acid nanoparticles	HEp-2 cells [89]
Lung carcinoma	Inhalable nanocomposites	A549 cells, mice [112]
	Lipid nanocarriers	A549 cells, mice [113]
	Inhalable nanocomposites	A549 cells, mice [58]
	Polymeric nanoparticles	A549 cells, mice [114]
Pancreatic cancer	Polymeric nanoparticles	PANC-1 cells, mice [115,116]
Retinoblastoma	Protein nanoparticles	Y79 cells [117]
**Other pathologies**		
Alzheimer	Protein nanoparticles	In silico [118]
	Polymeric nanoparticles	16HBE, SH-SY5Y cells, mice [119]
	-	Review [120]
	Polymeric nanoparticles	bEnd3 cells, mice, rats [121]
Amyotrophic lateral sclerosis	Polymeric nanoparticles	Review [122]
Dental caries	Liposomes	Rats [123]
Leishmaniosis	Polymeric nanoparticles	Mice [124]
Infection by Clostridioides	Polymeric nanoparticles	Caco-2, Vero cells [6]
Infection by *Helicobacter pylori*	Nanocrystals	Mice [125]
HIV	Nanosuspensions	U-937 cells, rats [126]
	Protein nanoparticles	SupT1, HL2/3 cells [127]
Infection by papilloma virus	Transferosomes	Hela cells [128]
Infection by *Toxoplasma* *gondii*	Protein nanocapsules	J7741 cells, mice [129]
Infection by Plasmodium berghei	Polymeric nanoparticles	Mice [130]
Osteoarthritis	Polymeric nanocarriers	Mice [131]
Parkinson	Protein nanoparticles	BCEC cells, rats [132,133]
	Polymeric nanoparticles	bEnd.3 cells, mice [134]
	Polymeric nanoparticles	SH-SY5Y, 16HBE cells, mice [135]
	Polymer, solid lipid nanoparticles, liposomes, exosomes	Review [136]
	Polymeric nanoparticles	SH-SY5Y, 16HBE cells, rats [137]
	Phosphorus nanosheets	SH-SY5Y, bEnd.3 cells, mice, rats [138]
Tendinitis	Polymeric nanoparticles	Tenocytes, rats [139]

**Table 2:** Studies regarding the use of lactoferrin in different systems for applications in food technology.

System	Other Molecules Combined	Activity
**Composite edible and active films**		
Chitosan films	Glycomacropeptide	Water vapour, oxygen and carbon dioxide permeability decrease [144]
	Lysozyme	Antimicrobial activity against *Listeria monocytogenes* and *Escherichia coli* O157:H7 [145]
Cellulose films	Lysozyme	Antimicrobial activity against *Escherichia, Listeria* and natural microbiota in veal meat [146]
	--	Antimicrobial activity against *Escherichia coli* and *Staphylococcus aureus* [147,148]
Starch films	Lysozyme	Antimicrobial activity against *E.coli* and coliform microbiota of pork minced meatLard oxidation reduction [149]
Gelatin-based films with chitosan nanoparticles	Quercetin	Antimicrobial activityAntioxidantFilm degradation improvement [150]
Polyethylene microtubes	Lactoferricin B	Antimicrobial activity against *Pseudomonas* strains responsible for casein hydrolysis and cheese pigmentation in mozzarella [151]
**LF nanoparticles**		
	Cichoric acid (CA)	Antioxidant [104]
	Pectin and curcumin	Antioxidant [152]
	Iron	Iron carrier [153]
		Functional ingredients in commercial products [64]
**Emulsions**		
	Resveratrol	Emulsion stability and antioxidant activity [44]
	WPI or whey protein hydrolysates with oil mixture	Ingredient in powder formula to mimic fat composition of human milk [34]
		To deliver active compounds [43,45]
**Coacervates**		
Whey proteins		Non-specified [28]
	B9 vitamin	For functional foods [29,30]
		Food systems, bioactive encapsulation [25,32]
Caseins		To deliver food ingredients and bioactive compounds [37,38,39,40]
Pea protein isolate (PPI)		Food applications [47]
Soy protein isolate (SPI)		Increase the thermal stability of LF [21]
**Gels**		
Whey protein isolate (WPI)		Improvement of gelled protein products [31]
GMP	Curcumin and caffeine	Encapsulation to deliver compounds [69]
Polysaccharides		Food additive [54]
		To deliver compounds [5]
		To encapsulate essential oils [52]
		To encapsulate probiotics [55]

## Data Availability

Data sharing is not applicable to this article.
